# Expectations of Anesthesiology and Intensive Care Professionals Toward Artificial Intelligence: Observational Study

**DOI:** 10.2196/43896

**Published:** 2023-06-12

**Authors:** Jan Andreas Kloka, Sophie C Holtmann, Elina Nürenberg-Goloub, Florian Piekarski, Kai Zacharowski, Benjamin Friedrichson

**Affiliations:** 1 Department of Anaesthesiology, Intensive Care Medicine and Pain Therapy University Hospital Frankfurt Goethe University Frankfurt Germany; 2 Chair for Special Education V – Education for People with Behavioural Disorders Faculty of Human Sciences University of Wuerzburg Wuerzburg Germany

**Keywords:** anesthesiology, artificial intelligence, health care, intensive care, medical informatics, technology acceptance, Europe-wide survey

## Abstract

**Background:**

Artificial intelligence (AI) applications offer numerous opportunities to improve health care. To be used in the intensive care unit, AI must meet the needs of staff, and potential barriers must be addressed through joint action by all stakeholders. It is thus critical to assess the needs and concerns of anesthesiologists and intensive care physicians related to AI in health care throughout Europe.

**Objective:**

This Europe-wide, cross-sectional observational study investigates how potential users of AI systems in anesthesiology and intensive care assess the opportunities and risks of the new technology. The web-based questionnaire was based on the established analytic model of acceptance of innovations by Rogers to record 5 stages of innovation acceptance.

**Methods:**

The questionnaire was sent twice in 2 months (March 11, 2021, and November 5, 2021) through the European Society of Anaesthesiology and Intensive Care (ESAIC) member email distribution list. A total of 9294 ESAIC members were reached, of whom 728 filled out the questionnaire (response rate 728/9294, 8%). Due to missing data, 27 questionnaires were excluded. The analyses were conducted with 701 participants.

**Results:**

A total of 701 questionnaires (female: n=299, 42%) were analyzed. Overall, 265 (37.8%) of the participants have been in contact with AI and evaluated the benefits of this technology higher (mean 3.22, SD 0.39) than participants who stated no previous contact (mean 3.01, SD 0.48). Physicians see the most benefits of AI application in early warning systems (335/701, 48% strongly agreed, and 358/701, 51% agreed). Major potential disadvantages were technical problems (236/701, 34% strongly agreed, and 410/701, 58% agreed) and handling difficulties (126/701, 18% strongly agreed, and 462/701, 66% agreed), both of which could be addressed by Europe-wide digitalization and education. In addition, the lack of a secure legal basis for the research and use of medical AI in the European Union leads doctors to expect problems with legal liability (186/701, 27% strongly agreed, and 374/701, 53% agreed) and data protection (148/701, 21% strongly agreed, and 343/701, 49% agreed).

**Conclusions:**

Anesthesiologists and intensive care personnel are open to AI applications in their professional field and expect numerous benefits for staff and patients. Regional differences in the digitalization of the private sector are not reflected in the acceptance of AI among health care professionals. Physicians anticipate technical difficulties and lack a stable legal basis for the use of AI. Training for medical staff could increase the benefits of AI in professional medicine. Therefore, we suggest that the development and implementation of AI in health care require a solid technical, legal, and ethical basis, as well as adequate education and training of users.

## Introduction

Artificial intelligence (AI) describes computer systems that simulate aspects of human intelligence, such as learning, logical thinking, and problem-solving. In this sense, AI is not a single technology but represents behaviors engendered by a set of computational models, processes, and algorithms. More recently, powerful computer hardware and the availability of vast amounts of data (big data) have accelerated the progress of AI. Established AI-based applications in industry and business are designed to ensure peak performance and minimize room for error [1]. AI is poised to transform our societies, daily lives [2], and health systems [3]. Nevertheless, the medical sector is still hesitating to broadly exploit these opportunities [4,5], even though numerous AI-based applications have been reported [6-11].

Intensivists must constantly assess a vast flow of information to make a diagnosis and decide on a treatment. According to an article by Morris [12], clinical professionals in intensive care units (ICUs) must permanently capture, classify, and contextualize up to 236 parameters, which far exceed human cognitive abilities. Thus, especially clinicians affiliated with ICUs could profit from novel AI-based applications, as numerous vital parameters are constantly collected for each patient and provide a reliable basis for computational support [13]. Notably, while AI could provide valuable assistance for clinicians in data-intensive environments, it must overcome numerous challenges to address the concerns of medical staff for robust implementation at points of care. Technical aspects, such as poor data quality and interoperability, low levels of collaboration between different stakeholders, the largely missing legal and ethical framework, and the lack of advanced education for users, were cited as major barriers to the deployment of AI in the medical field [4,5,14]. Accordingly, numerous algorithms developed for the ICU remain in the stage of scientific publication and do not reach clinical implementation [15,16]. Rogers Model of Innovation Acceptance (Diffusion of Innovations Theory) and the NASSS (nonadoption, abandonment, scale-up, spread, and sustainability) framework are established and well used to understand and facilitate the adoption and implementation of new technologies and innovations in various settings, including health care [17,18].

This study investigates the expectations toward AI applications among European intensive care staff and anesthesiologists. Based on the Diffusion of Innovations theory, this project aims to derive valuable conclusions to guide the development and implementation of AI applications in the ICU.

## Methods

### Overview

A web-based questionnaire was designed for the cross-sectional and observational study based on the established analytical model of acceptance of innovations by Rogers. The model (Diffusion of Innovations Theory) is used to understand and facilitate the adoption and implementation of new technologies and innovations in various settings, including health care [19].

The survey aimed to describe the acceptance of AI applications among European health care professionals as well as the benefits and drawbacks they see and expect from AI implementation in their work environment (Multimedia Appendix 1). The following demographic variables were assessed: gender, age, working position, medical specialization, and country of employment.

The questionnaire was tested and modeled in an initial test phase by 5 physicians for practicability and content. The survey was open to all European health care professionals (physicians and nurses working in all positions in anesthesiology and intensive care). Due to the chosen distribution method, mainly anesthesiologists and intensive care physicians were reached.

The survey was implemented for 2 months, starting on March 11 and lasting until May 9, 2021. The survey was distributed through the European Society of Anaesthesia and Intensive Care (ESAIC) to its members by email with a link to the survey and an invitation to participate. In addition, the link was published on the home page of the European Union (EU)–funded project “ENVISION” [20].

### Ethical Considerations

In a declaration of consent, participants were informed about the study, data privacy, and anonymous data collection. The Ethics Committee of the University Hospital Frankfurt, Frankfurt, Germany, waived the need for ethical committee approval for this study (Chairperson Prof Dr Harder, Ref No. 2022-766, 28.4.22). Due to the anonymous data collection, no conclusions can be drawn about individual participants. Therefore, the European General Data Protection does not apply. The study was planned in concordance with the CROSS (Checklist for Reporting of Survey Studies) guideline [21]. There was no compensation for the voluntary participation.

To get the status quo in Europe within the currently working health care professionals, questionnaires from participants aged 25-67 years, representing the range of standard ages between completed education and retirement in Europe, were included. Questionnaires that were incompletely filled were excluded.

All statistical analyses were conducted using SPSS 26.0.0.1 [22]. An alpha level of *P*=.05 was set for all analyses. Bonferroni correction was applied for multiple comparisons. Statistical relationships were detected by product-moment correlation coefficients (1-tailed). Continuous data were examined with univariate and multivariate analyses of covariance, using simple contrasts to test for group differences. For statistical calculations, countries were grouped according to the geographic scheme of the statistics division of the United Nations [23].

Categorical variables were analyzed by the chi-square independence test. Effect sizes between 0.01 and 0.039 were interpreted as small, between 0.06 and 0.11 as medium, and from 0.14 as strong [24,25].

## Results

### Responders’ Characteristics

Within 2 months, 728 questionnaires were received, of which 27 had to be excluded due to missing data. In total, 701 participants from all 27 EU members and other non-European states working at different positions in the health care sector were included. The participants had a mean age of 45.86 (SD 10.32, range 25-67) years. The gender distribution was 299 (43%) female and 402 (57%) male, with representative proportions for the different working positions (Table 1). A total of 57 (8%) participants were assigned to northern, 165 (24%) to western, 82 (12%) to eastern, and 178 (25%) to southern Europe. Non-EU countries were classified as “other” (219/701, 31%) (Table S1 in Multimedia Appendix 2). Due to the distribution route and in line with the target group, most participants were affiliated with anesthesiology or intensive care medicine (691/701, 99%). More than two-thirds of all participants stated that they have not been in contact with AI up to now (436/701, 62%), and 265 (38%) have been in contact with AI in 1 or more than one field: private (142/265, 54%), professional (132/265, 50%), scientific (80/265, 30%), or other fields (17/265, 6%). The results are structured into 5 key points, which are investigated and discussed below.

**Table 1:** Demographics. Frequency among the respective gender. Gender distribution of participants among different working positions. Both genders of participants included into the data set are equally represented among all working positions.

			Medical working position											Total, n (%)
			Resident, n (%)		Specialist, n (%)		Senior consultant, n (%)		Chief physician, n (%)		Other, n (%)			
**Gender**														
	Female (n=299)	42 (14)		140 (47)		83 (28)		28 (9)		6 (2)		299 (100)		
	Male (n=402)	46 (11)		166 (41)		130 (32)		54 (13)		6 (1)		402 (100)		

### Impact of Gender on the Awareness of AI

There was no significant relationship between sex and awareness of AI (*χ*^2^_1_=3.02; *P*=.08) (Table S2 in Multimedia Appendix 2). The distribution of female and male participants was unrelated to the age or the working position (*χ*^2^_4_=6.001; *P*=.20), thus correctly reflecting gender-specific results (Table 1)

### Regional Differences in the Awareness of AI

There was no significant relationship between the region and the awareness of AI (*χ*^2^_4_=6.495; *P*=.17), even the calculation without the non-EU regions results in no differences (*χ*^2^_1_=2.706; *P*=.10) (Table 2).

**Table 2:** Contact with artificial intelligence (AI) according to regions. Absolute number of participants (N=701). Contact with AI in European regions. Grouping the participants into regions allowed significant statistical evaluation of the results.

		Northern Europe (n=57), n (%)	Western Europe (n=165), n (%)	Eastern Europe (n=82), n (%)	Southern Europe (n=178), n (%)	Other (n=219), n (%)
**Previous contact with AI**						
	Yes	22 (39)	74 (45)	34 (42)	62 (35)	73 (33)
	No	35 (61)	91 (55)	48 (58)	116 (65)	146 (67)

### Benefits and Difficulties of AI in the Professional Field

**Figure 1:**
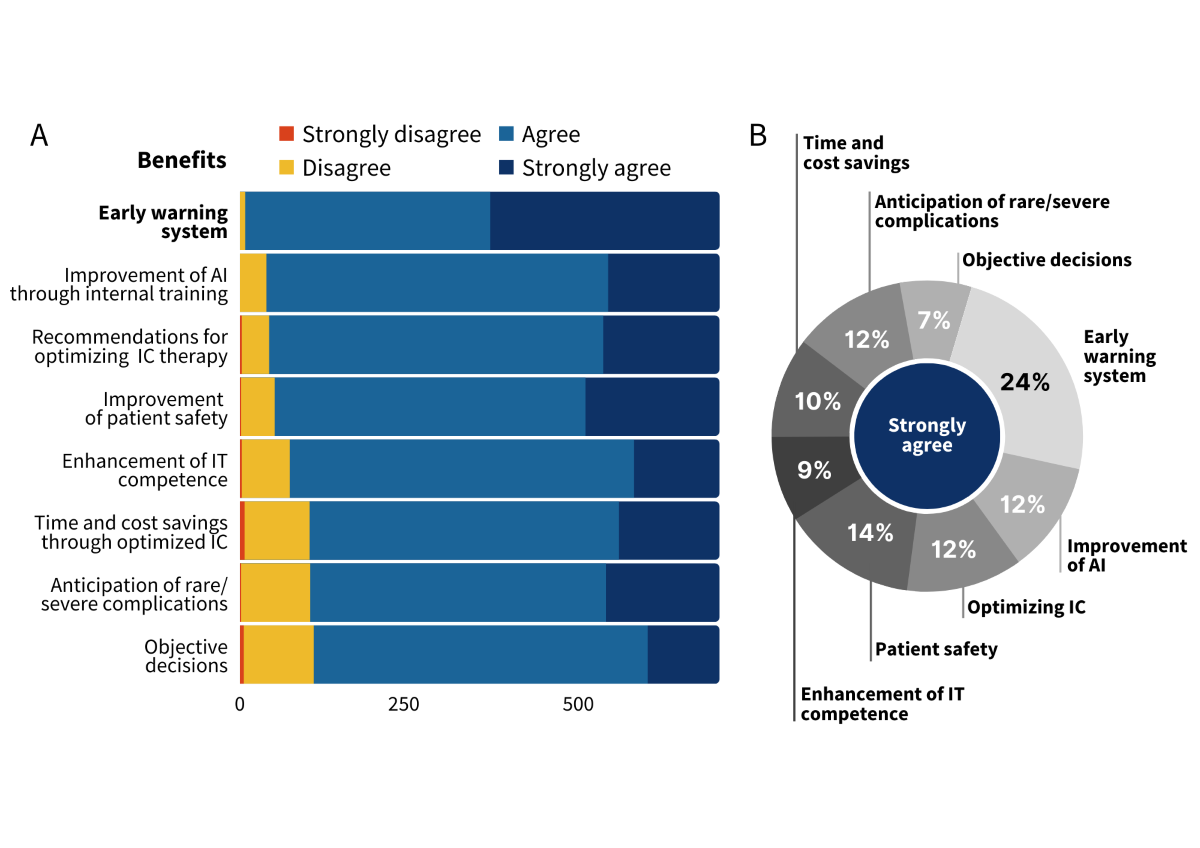
Potential benefits of AI. (A) Distribution of the votes for rating potential benefits of AI applications by all included participants. The numbers indicate the total number of votes. Potential applications are sorted by the descending total number of votes agreeing or strongly agreeing with the expected benefit. (B) Distribution of “strongly agree” votes among the potential benefits of AI in health care (N=1407). An AI-based early warning system is expected to be most useful in European health care. Among all benefits, the participants strongly agreed that an AI-based early warning system would be the most beneficial. Objective decisions made by AI are least expected to improve work in intensive care units. AI: artificial intelligence; IC: intensive care.

The descriptive analysis showed that participants mostly agreed with the benefits of AI technology in the medical sector. The highest benefit (most agreeing participants, Table S3 in Multimedia Appendix 2) is seen in early warning systems, which 335 of 701 (47%) participants even strongly agreed with (corresponds to 335/1407, 24% of all strongly agreeing votes), followed by the improvement of AI through internal training (163/701, 23% of all participants, and 163/1407, 12% of all strongly agreeing votes) and the optimization of intensive care therapy (170/701, 24% of all participants, and 170/1407, 12% of all strongly agreeing votes) (Figure 1).

**Figure 2:**
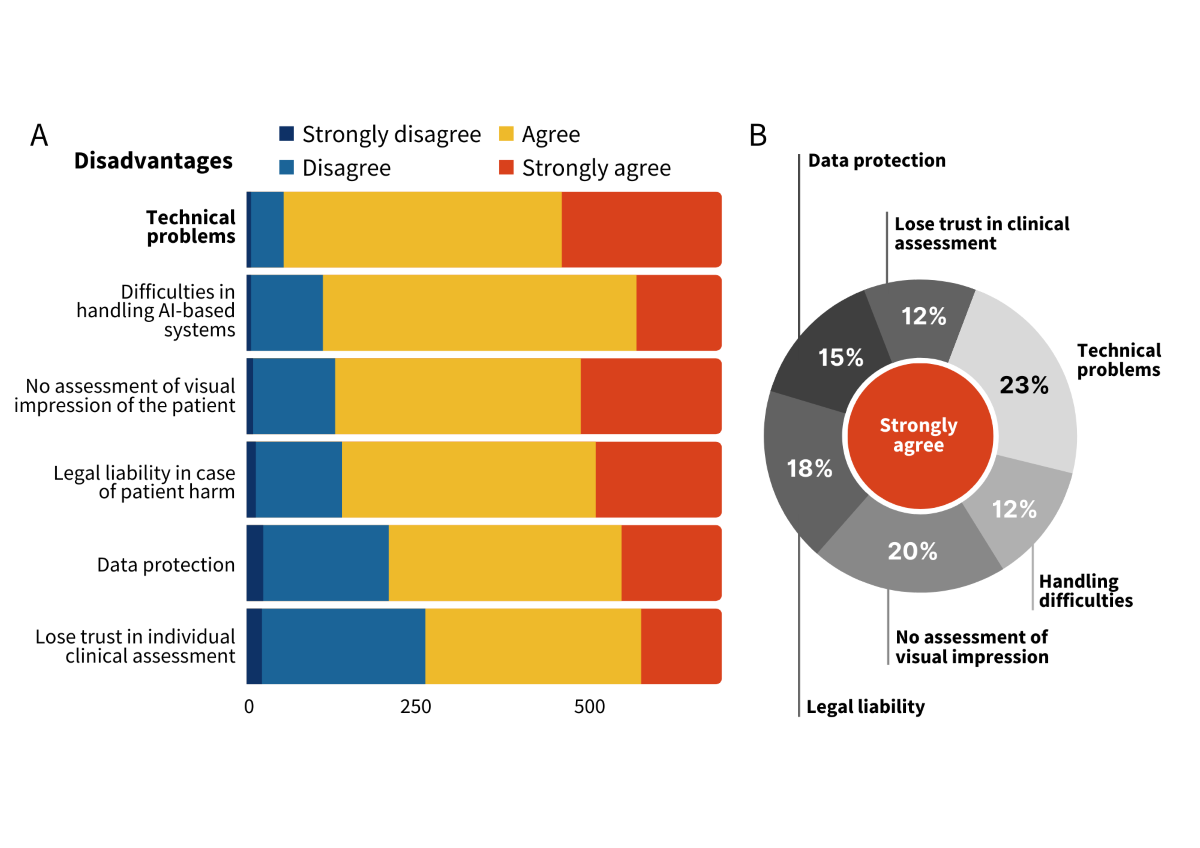
Potential difficulties of AI. (A) Distribution of the votes for rating potential disadvantages of AI applications by all included participants. The numbers indicate the total number of votes. Potential risks are sorted by the descending total number of votes agreeing or strongly agreeing with the expected disadvantage. (B) Distribution of strongly agreeing votes among the potential disadvantages of AI in health care (N=1023). Among all disadvantages, most participants strongly agreed to expect technical problems with AI-based systems. In contrast, the participants do not expect that individual clinical assessment will lose trust in intensive care units due to an increased presence of AI technology. AI: artificial intelligence.

The largest perceived difficulties (most agreeing participants, Table S4 in Multimedia Appendix 2) were technical problems (236/701, 34% of all participants and 236/1023, 23% of all strongly agreeing votes), difficulties in handling AI-based systems (126/701, 18% of all participants and 126/1023, 12% of all strongly agreeing votes), and the inability of AI to visually assess the patient (208/701, 30% of all participants and 208/1023, 20% of all strongly agreeing votes) (Figure 2).

Notably, there was a significant 1-tailed correlation between benefits and disadvantages (*r*=0.141; *P*<.001). The higher participants rated the benefits, the lower they rated the disadvantages. According to Cohen [24], it is a rather weak effect.

### Impact of Previous Contact With AI on the Assessment of Benefits and Difficulties in the Professional Field

Multivariate analysis of covariance (MANCOVA) revealed a significant main effect on the assessment of AI and previous contact with AI (Pillai trace *F*_2,694_=7.1; *P*=.001; η_p_^2^=0.02). The difference remains significant if the covariates of sex, region, medical position, and age are controlled. The planned contrast revealed a significant difference in the assessment only for the benefits (Pillai trace *F*_1,695_=13.85; *P*<.001; η_p_^2^=0.02). Participants who were in contact with AI before, evaluated each listed benefit higher (mean 3.22, SD 0.39) than participants who stated no previous contact with AI (mean 3.01, SD 0.48). Moreover, there is a contrast between participants who have had experience with AI in a professional context and those who have not yet used AI at work in terms of their evaluation of the benefits (Pillai trace *F*_8,592_=28.94; *P*<.001; η_p_^2^=0.28). Those who have had experience with AI rate the benefits of AI overall higher than those who have not had experience with it. Age was the only covariate that also had a significant influence (Pillai trace *F*_8,592_=6.5; *P*=.03; η_p_^2^=0.08) with a negative correlation; the older the participants, the lower the perceived benefits of AI applications.

## Discussion

### Background

We assessed the advantages and difficulties that physicians in European critical care and anesthesiology expect from implementing AI-based applications in their work. Early warning systems have a clear edge among all the benefits assessed by physicians, uncovering a largely underserved clinical need. In line with challenges highlighted in previous research [4,5,14,15,26,27], our survey shows that intensivists and anesthesiologists anticipate a high potential for technical problems and legal issues related to defining liability and ensuring data protection. Further, medical staff would benefit from advanced education and training to mitigate handling difficulties and improve IT competence.

### Gender Has No Significant Impact on the Awareness of AI

The previously reported considerable gender gap in digital inclusion in the emerging countries among the G20 economies [28] is not reflected in our pool of highly educated individuals in the EU. The gender distribution of our participants was uniform among the different European regions and working positions, with a slight insignificant overweight in male senior consultants and chief physicians (4% points, respectively). Thus, we conclude that awareness of AI is likewise popular among female and male clinicians.

### Regional Differences in the Digitalization of the Private Sector Are Not Reflected in AI Acceptance Among Health Care Workers

Europe is a heterogeneous continent in many ways. The digitalization of European companies is way behind the digital adoption in the United States, according to the European Investment Bank Investment Survey (EIBIS) [29]. Thus, only 63% of firms in Europe (vs 73% in the United States) have implemented sophisticated digital technologies like advanced robotics, 3D printing, AI, or the internet of things. The adoption rate differs widely in individual EU countries. While in Sweden, Finland, Spain, and Denmark, more than 70% of firms have introduced advanced digital technologies, in Greece, France, and Poland, the adoption rate is below 54% [29]. Surprisingly, the awareness of AI applications among health care workers is equally distributed among the 4 European regions and beyond the EU in our survey. The results of this study suggest that AI-based tools, once developed and implemented, will be widely and uniformly accepted in ICUs in all European regions.

### Physicians Anticipate Technical Difficulties and Miss a Stable Legal Fundament for the Use of AI

In line with the heterogeneous adaptation of advanced digital technologies in the EU, the weak digital infrastructure is reported to be a barrier to investments in many member states [29]. In our survey, 646 (92%) participants expected technical problems with AI applications (Figure 2, Table S4 in Multimedia Appendix 2). The lack of digital infrastructure in hospitals and health care administrations limits the introduction of new technologies, and governments should show receptiveness. In 2020, the US Food and Drug Administration listed the biggest advantages of health care digitalization, which were reduced inefficiencies, reduced costs, increased quality, and more personalized medicine for patients [30]. The European Commission is currently running programs to support digitalization in the health care sector [31], including the ambitious European Health Data Space project [32]. Problems anticipated from the lack of a legal framework for the development and use of medical AI are strongly related to critical technical questions like data availability, ownership, security, and interoperability. Critical care physicians named unclear legal liability (560/701, 80%) and data protection issues (491/701, 70%) as potential challenges of AI-based applications in their working environment. To unleash the potential of AI at the bedside, law and technology must work closely together [5,26]. Thus, we appreciate the efforts of the European Commission to create a technical basis and stimulate the development of a consistent legal and ethical foundation for AI usage in the medical sector across the EU.

### Advanced Training for Medical Staff Might Enhance the Benefits of AI Used in Professional Medicine

In 2019, a web-based survey among fellows of the ophthalmology, radiology, radiation oncology, and dermatology colleges in Australia and New Zealand revealed that the majority of participants (71%) expect an improvement in medicine by AI within the next decade. Key advantages were named to be improved disease screening and streamlining of monotonous tasks, while health care outsourcing to technology companies and implications for medical liability were the biggest concerns [33]. The majority of European radiology students believed that AI could be able to detect pathologies (83%) and would in the future improve radiology (86%) [34]. Likewise, doctors and the public in Japan are enthusiastic about the use of AI-driven approaches in medicine [35].

We have been able to reveal a strong dependence of the evaluation of AI applications on previous contact with AI. The more AI-based applications were used, the more their presumed benefits outweigh the disadvantages, as judged by European health care professionals surveyed in our study. This is in line with the Rogers Model of Innovation Acceptance, which identifies 5 stages of innovation acceptance (knowledge, persuasion, decision, implementation, and confirmation) and describes how individuals and organizations progress through the adoption process. Notably, we cannot assess if all participants are aware of AI in digital systems supporting their daily lives and work routines. According to our data, only 265 (38%) of the 701 participants have been in contact with AI. This is a questionable finding since AI has become an everyday application used to unlock smartphones, personalize social media accounts, newsfeeds, and search engines. Strikingly, we focused on anesthesiologists and intensivists, who are working in a data-intensive field and thus are expected to be most aware of advanced technology and computation-assisted medical applications. This discrepancy raises the potential need to increase awareness and intensify education and advanced training in the field of computer technologies for medical staff in all working positions in Europe.

For the development of technological innovation projects, it is important to consider the benefits and difficulties future users see in AI.

Consistent with previous surveys among medical students, most of our participants expect multiple benefits from the implementation of AI into the health care sector. According to Khullar et al [36], patients also have positive views about AI’s ability to improve care. The authors conclude that patients may benefit from education on how AI can assist in health care. We can underline the need for education not only for patients but also for clinical users to address concerns.

We identified the common advantages and possible difficulties as seen by potential users. Notably, 588 (84%) participants anticipate difficulties in handling AI applications (Figure 2, Table S4 in Multimedia Appendix 2). Consistently, 628 (90%) participants agreed that the use of AI at work would enhance their IT competence (Figure 1, Table S3 in Multimedia Appendix 2).

Rogers Model of Innovation Acceptance and the NASSS framework are highly relevant for the implementation of innovative technologies in complex and interdependent environments like health care [17,18]. One of the relevant domains is the adopter system, namely medical staff, patients, and lay caregivers [18]. Meaningfulness and explainability were pointed out as major considerations for health care providers in the implementation of AI-based decision support [27]. It is therefore essential to address the demands and concerns of health care workers to ensure better adaptation, and thus, acceptance of costly new technologies. Our findings emphasize the requirement for advanced training programs for clinicians as well as the development of intuitive user interfaces for AI applications to meet the needs of health care professionals.

### Limitations

Web-based surveys are growing in popularity. However, there are limitations that need to be addressed. Web-based surveys are distributed through different digital channels without the possibility of describing the population that accessed and responded to the survey. This survey has been distributed through an email distribution list to all ESAIC members, and the link to the survey has been published on our ENVISION website. Therefore, the Europe-wide response is not representative. There is no standardized and evaluated questionnaire on this topic; therefore, an adapted analytic model of acceptance of innovations by Rogers was used [19]. The survey was conducted during the COVID-19 pandemic, with intensive care stations overrun by patients all over Europe. That is why the survey was limited to a maximum processing time of under 10 minutes, which hindered a closer query on further detail. Despite the short and easy-to-answer survey, only 728 (8%) of the 9294 invitees answered the survey. This response rate is within the lower limits of a web-based survey [37]. This might be explained by the high number of patients with COVID-19 during this time and the consecutive high workload for medical professionals. Though the survey potentially harbors a nonresponse bias, we believe that the conclusions drawn are well supported by the existing literature.

### Conclusion

This Europe-wide, web-based survey has shown that anesthetists and intensivists are open to AI applications in their professional field. Numerous benefits for staff and patients are expected by the participants. Regional differences in the digitalization are not reflected in the acceptance of AI among health care professionals. The participants anticipate technical difficulties and lack a stable legal basis for the use of AI. For AI systems to be successfully deployed in European ICUs for the benefit of staff and patients, we emphasize several action points: (1) financial and political measures to reduce technical barriers and create an equivalent level of digitization in European hospitals; (2) incentives for collaboration among all stakeholders to develop meaningful and user-friendly applications; (3) the elaboration of a strong legal framework that makes scientific research and the use of AI safe and trustworthy; and (4) adequate education and training of medical staff.

## Appendix

Multimedia Appendix 1 Spotlight questionnaire.

Multimedia Appendix 2 Supplement tables.

## Abbreviations

AI: artificial intelligence
CROSS: Checklist for Reporting of Survey Studies
EIBIS: European Investment Bank Investment Survey
ESAIC: European Society of Anesthesiology and Intensive Care
EU: European Union
ICU: intensive care unit
MANCOVA: multivariate analysis of covariance
NASSS: nonadoption, abandonment, scale-up, spread, and sustainability

## Data Availability

The data sets generated and analyzed during this study are available from the corresponding author on reasonable request.
