# Supplementary material for: Expectations of Anesthesiology and Intensive Care Professionals Toward Artificial Intelligence: Observational Study
Source: JMIR Form Res. 2023 Jun 12;7:e43896. doi: 10.2196/43896 (PMC10337415; doi:10.2196/43896)
Supplement: Multimedia Appendix 1 [file formative_v7i1e43896_app1.pdf]

Page 01

Intro

Dear participants,

The use of artificial intelligence (AI) assistance systems is becoming increasingly important. AI is also finding its way into the medical sector and can offer great opportunities for clinical users.The study intends to measure the use of artificial intelligence and its acceptance among medical professionals.

In cooperation with the European Society of Anaesthesiology and Intensive Care (*ESAIC*), we are developing an AI-based plug-and-play device to support the treatment of COVID-19 patients in intensive care. The project is supported by the European Union (for more information see: www.envision-icu.eu).

By participating in this survey, you allow us to address your needs and concerns as potential users.

Filling out a questionnaire is a lot to ask for in the current times. Therefore, we designed a short questionnaire with a completion time of **about 5 minutes**.

The survey is completely anonymous, the data will be treated confidentially and will only be evaluated for scientific purposes. Participation in the survey is voluntary.

We thank you in advance for your support!

Page 02

Alter

How old are you?

Age: ____ years

Page 03
Sex

Sex?

- ○ Female
- ○ Male
- ○ Various

Page 04
Land

In which country do you work?

[Please choose]

Page 05
MedPosition

What is your medical position?

I am a [Please choose]

in the field of [Please choose]

Page 06
YN Kontakt

Have you ever been in contact with artificial intelligence (AI)?

- ○ Yes
- ○ No

In what context have you been involved with artificial intelligence (AI)?

- ☐ In private environment
- ☐ In professional field
- ☐ In the scientific field
- ☐ Other field

Please rate the statements on the use of artificial intelligence (AI) according to your personal experience in the medical field.

	strongly disagree	disagree	agree	strongly agree
I can cope well with changes in the workplace	○	○	○	○
Innovative systems support my daily work routine	○	○	○	○
Technical innovations improve my work	○	○	○	○
I am supporting AI implementations in my daily work routine	○	○	○	○
My colleagues support the implementation of AI in our work routine	○	○	○	○
I trust AI recommendations for action in intensive care treatment (or my medical field)	○	○	○	○
I fear that medical competence will be increasingly restricted by AI implementations	○	○	○	○
I have already informed myself about AI systems	○	○	○	○

Please rate the statements on the use of artificial intelligence (AI) according to your personal expectations in the medical field.

	strongly disagree	disagree	agree	strongly agree
I can cope well with changes in the workplace	○	○	○	○
Innovative systems support my daily work routine	○	○	○	○
Technical innovations improve my work	○	○	○	○
I would support AI implementations in my daily work routine	○	○	○	○
My colleagues would support the implementation of AI in our work routine	○	○	○	○
I would trust AI recommendations for action in intensive care (or my medical field)	○	○	○	○
I fear that medical competence will be increasingly restricted by AI implementations	○	○	○	○
I have already informed myself about AI systems	○	○	○	○

What benefits can be expected from the use of artificial intelligence (AI) in the medical field?

	strongly disagree	disagree	agree	strongly agree
Objective decisions	○	○	○	○
Early warning system	○	○	○	○
Recommendations for optimizing intensive care therapy	○	○	○	○
Time and cost savings through optimized intensive care treatment	○	○	○	○
Continuous improvement of AI through internal training	○	○	○	○
Enhancement of IT competence	○	○	○	○
Improvement of patient safety	○	○	○	○
Anticipation of rarer/less severe complications through AI	○	○	○	○

What difficulty(ies) do you think could arise from an artificial intelligence (AI) implementation at your work?

	strongly disagree	disagree	agree	strongly agree
Difficulties in handling AI based systems	○	○	○	○
Technical problems	○	○	○	○
Data protection	○	○	○	○
Legal liability in case of accident/patient harm	○	○	○	○
Lose trust in individual clinical assessment	○	○	○	○
AI does not have the ability to assess the visiual impression of the patient	○	○	○	○

Thank you for completing this questionnaire!

We would like to thank you very much for helping us.

Your answers were transmitted, you may close the browser window or tab now.

Dr. Jan Kloka, Dr. Benjamin Friedrichson, https://www.envision-icu.eu/,

Department of Anaesthesiology, Intensive Care Medicine and Pain Therapy, University Hospital Frankfurt, Germany
