# Supplementary material for: Expectations of Anesthesiology and Intensive Care Professionals Toward Artificial Intelligence: Observational Study
Source: JMIR Form Res. 2023 Jun 12;7:e43896. doi: 10.2196/43896 (PMC10337415; doi:10.2196/43896)
Supplement: Multimedia Appendix 2 [file formative_v7i1e43896_app2.docx]

### Supplement Tables

**Table S1: Distribution of participants among the European regions.**

|  | **Absolute number** | **Frequency in %** |
| --- | --- | --- |
| **Northern Europe** | 57 | 8.1 |
| **Western Europe** | 165 | 23.5 |
| **Eastern Europe** | 82 | 11.7 |
| **Southern Europe** | 178 | 25.4 |
| **Non-EU countries** | 219 | 31.2 |

**Table S2: Contact with AI among female and male participants.**

|  | | absolute number of participants, N = 701 | |
| --- | --- | --- | --- |
|  |  | **Previous contact with AI** | |
|  |  | **Yes** | **No** |
| **Gender** | **Female** | 102 | 197 |
|  | **Male** | 163 | 239 |
| ∑ | | 265 | 436 |

**Table S3: Number of votes for potential benefits of AI technology in healthcare.**

|  | total number of votes | | | | ∑ (*n* = 701) | |
| --- | --- | --- | --- | --- | --- | --- |
| **benefit** | **strongly disagree** | **disagree** | **agree** | **strongly agree** | **agree** | **disagree** |
| early warning system | 0 | 8 | 358 | 335 | 693 | 8 |
| improvement of AI through internal training | 0 | 39 | 499 | 163 | 662 | 39 |
| recommendations for optimising intensive care therapy | 3 | 40 | 488 | 170 | 658 | 43 |
| improvement of patient safety | 2 | 49 | 454 | 196 | 650 | 51 |
| enhancement of IT competence | 3 | 70 | 503 | 125 | 628 | 73 |
| time and cost savings through optimised intensive care | 7 | 95 | 452 | 147 | 599 | 102 |
| anticipation of rare/severe complications | 2 | 101 | 432 | 166 | 598 | 103 |
| objective decisions | 6 | 102 | 488 | 105 | 593 | 108 |
| **∑** | **23** | **504** | **3674** | **1407** | **5081** | **527** |

**Table S4: Number of votes for potential disadvantages of AI technology in healthcare.**

|  | total number of votes | | | | ∑ (*n* = 701) | |
| --- | --- | --- | --- | --- | --- | --- |
| **disadvantage** | **strongly disagree** | **disagree** | **agree** | **strongly agree** | **agree** | **disagree** |
| technical problems | 7 | 48 | 410 | 236 | 646 | 55 |
| difficulties in handling AI-based systems | 7 | 106 | 462 | 126 | 588 | 113 |
| no assessment of visual impression of the patient | 10 | 121 | 362 | 208 | 570 | 131 |
| legal liability in case of patient harm | 14 | 127 | 374 | 186 | 560 | 141 |
| data protection | 25 | 185 | 343 | 148 | 491 | 210 |
| lose trust in individual clinical assessment | 23 | 241 | 318 | 119 | 437 | 264 |
| **∑** | **86** | **828** | **2269** | **1023** | **3292** | **914** |
